# Examining the Benefits and Barriers of Instructional Gardening Programs to Increase Fruit and Vegetable Intake among Preschool-Age Children

**DOI:** 10.1155/2017/2506864

**Published:** 2017-05-21

**Authors:** Kristen L. Davis, Lynn S. Brann

**Affiliations:** Department of Public Health, Food Studies and Nutrition, Syracuse University, Syracuse, NY, USA

## Abstract

Research exists on using instructional gardening programs with school age children as a means of improving dietary quality and for obesity prevention. This article examines the potential use of instructional gardens in childcare settings to improving fruit and vegetable intake in young children. A qualitative study was conducted with childcare providers. Participants (*n* = 20) were recruited via e-mails, letters, and follow-up phone calls. Interviews were recorded, transcribed, and coded to identify themes within two areas (1) childcare providers perceptions of children's fruit and vegetable consumption and (2) components necessary to initiate or improve instructional gardening programs. Themes associated with provider's perceptions of child fruit and vegetable consumption included benefits of consumption, willingness to try fruits and vegetables, meeting recommendations, and influence of the home and childcare environments on child eating. Benefits, barriers, and resources needed were identified as themes related to starting or improving instructional gardening programs. Benefits to gardening with preschoolers are consistent with those found in school-age populations. While several barriers exist, resources are available to childcare providers to address these barriers. Increased knowledge and awareness of resources are necessary to improve the success of gardening programs in the childcare setting with the goal of improving child diet quality.

## 1. Introduction

In an effort to increase fruit and vegetable (F/V) consumption among children, decrease the risk for obesity, and improve nutritional status, the implementation of school gardening programs has been examined. Previous research has shown that instructional gardens improve a child's ability to identify specific F/V and this increased awareness can help to increase his or her willingness to try and consume these foods more often [1]. Additionally, repeated positive experiences with F/V, increasing accessibility and availability of them, and gaining knowledge about where these foods come from through instructional gardening experiences have all been shown to increase preferences for and intake of F/V among children [2–6]. Recent gardening interventions were successful in improving the weight and nutritional status of young children by improving their intake of F/V as well as their level of physical activity [7].

Currently, most existing literature relating to school gardening programs focuses on school-age children and adolescents. While little literature exists investigating the impact of instructional gardening in a childcare setting, the research that has examined this has found success in increasing F/V consumption [6]. The lack of extensive previous research, combined with the need for this specific age group to be targeted for nutrition interventions to prevent childhood obesity, makes increasing research in this area a necessity. The age between 2 and 5 years old is extremely important to the development of a child, as children are growing rapidly and are developing a large amount of their food preferences [2].

The number of children under the age of 5 in the US who are cared for outside the home is approximately 12.5 million [8], making the childcare environment an influential factor in the food intake patterns of children. Although many childcare facilities are providing meals which follow the Dietary Guidelines, many children are still not meeting recommendations for several food groups, particularly F/V [9, 10]. This makes instructional garden use an opportunity to improve the diet quality of children.

There could be several reasons for the lack of program implementation using instructional gardens including actual or perceived barriers such as a lack of time, lack of volunteers, or lack of garden coordinators [11, 12]. Although there are many resources that can be utilized, childcare providers may be unaware that they exist [13, 14]. A summary report from The National Gardening Association, a source for grants used annually to fund instructional and community garden programs, showed that 33% of their funding was utilized by preschool or Head Start programs in 2013 [15]. While this is an increase from the 8% reported in 2011 [16], it appears this age group is still somewhat underutilizing these available resources.

The theoretical framework used to guide this study is the Ecological Systems Theory (EST) originally developed by Bronfenbrenner [17] and adapted by Birch and Ventura [18] to fit the context of childhood overweight and obesity. This theory was chosen because it uses a systems approach to understand the layers of influence on a child's consumption of F/V intake. The microsystem with the impact of childcare environment on children's diet and also the mesosystem with the perceived interactions of the family and childcare environments are of particular relevance. The child's consumption of F/V, the behavior targeted by this research study, is in the center of the model. The objectives of this research study were to assess childcare providers' perceptions of children's F/V intake and to examine the components that act as benefits and barriers, both perceived and actual, to implement instructional gardening programs in childcare settings as a means for increasing F/V intake.

## 2. Participants and Methods

A qualitative research design was employed with a convenience sample of participants.

### 2.1. Participant Inclusion and Recruitment

A county resource and referral agency for both childcare providers and parents was utilized to identify potential participants. Researchers obtained contact information for all licensed childcare providers. To be included in the study, the participant must have been a state-licensed childcare provider currently caring for children between the ages of 2 and 5 years old or be in the process of becoming licensed.

The first method of recruitment was a mass e-mail solicitation to all registered childcare providers. Three participants were recruited this way. The second method of recruitment was a solicitation letter mailed to randomly selected childcare providers, generating 163 names to receive recruitment letters. Five participants were recruited using this method. Next, follow-up phone calls were made to potential participants by whom four participants were recruited. The recruitment mailing and follow-up phone calls were repeated with a second round of randomly selected individuals resulting in four additional participants. Finally, four childcare providers were recruited through snowball sampling using referrals from other study participants. A total of 20 participants were recruited for this study. Approval was obtained from the Institutional Review Board prior to recruitment and data collection.

### 2.2. Data Collection

Data were gathered via structured interviews with participants. A total of five phone interviews and 15 in-person interviews were conducted. First, demographic questions were asked to gain information on the type of childcare setting. Children's dietary habits were discussed focusing on F/V intake. Finally, gardening activities were discussed including the benefits of and barriers to gardening. These questions differed among interviews depending on whether the childcare provider was currently using gardening activities or not. A list of structured interview questions is included in Table 1.

All interviews were recorded using a Digital Voice Recorder and were transcribed verbatim by the researcher.

### 2.3. Coding and Analysis

Each transcribed interview was coded using QSR NVivo 10 software. Coding was completed by separating answers to each interview question first related to childcare providers perceptions of children's F/V consumption and then components related to initiating and improving instructional gardening programs with young children. Data within these themes were labeled to generate codes. Data that were tangential and deemed unrelated to the main themes were removed from analysis. While one researcher was primarily responsible for coding the interviews, both researchers evaluated the themes and codes to come to agreement on the interpretation of the data.

## 3. Results

### 3.1. Demographic Information

The type of childcare setting, range in number of children attending, age of those children, and number of care providers employed at each facility are shown in Table 2.

### 3.2. Childcare Providers' Perceptions of Children's Fruit and Vegetable Consumption

Using the EST, childcare providers are potential influencers (at the microsystem and mesosystem levels) of children's dietary intake including their F/V intake. Themes that emerged in this area of childcare providers' perceptions of children's F/V intake included benefits of F/V consumption, willingness to try F/V, meeting F/V recommendations, and influences of the home and childcare environments on children's eating behaviors. Overall, participants felt that F/V consumption was very important for young children. Participants discussed the need for F/V consumption to provide important nutrients such as vitamins and minerals, variety in the diet, promoting independence in eating, and promoting digestive health. One provider stated, “I think fruits and vegetables offer a lot of diversity in terms of choices that kids can make themselves. Also, a lot of those kinds of foods are easy to eat and allow the children to be independent.”

Regarding willingness to try F/V, many providers felt that there was difficulty in getting children to try vegetables. Overall, childcare providers reported a greater willingness to try F/V among younger children as compared to older children. One provider stated, “[Younger children] don't have any preconceived notions of what it might taste like.” Another provider said “They tend to try it. Just exposure is a big thing. Just continuously exposing it, sometimes doing it a little differently.”

Providers discussed serving meals to children while in the childcare setting that met recommendations for F/V intakes based on nutrition guidelines. Although F/V were served, childcare providers expressed concerns about children not meeting recommendations. One stated, “We have a set schedule as to how much they have to consume per day. Obviously, you know, they don't always eat it, but they're served what they should be getting from us on a daily basis.”

The influence of the childcare environment and home environment was discussed in relation to child F/V consumption. Most participants felt that the childcare environment could offer a positive influence on eating behaviors due to aspects offered such as family-style dining, influence of teachers and peers, exposure to a greater variety of foods, and few substitutions offered at meal time. One provider stated, “I think it's important to have us all be together and all…I think having everyone eating the same thing makes a big difference in getting kids to try things they wouldn't otherwise try.” Providers had the impression that children were eating less healthy food while in their home environments due to hectic schedules and the tendency for parents to give in to “picky eaters.” A provider said, “I think most parents want their children to eat healthy, but I know that they are also at a different mindset than we are where they just, you know, kind of want low drama and maybe give in a little more than we do.” Overall, childcare providers discussed having a positive influence on the F/V intake of the children in their care.

### 3.3. Gardening in the Childcare Setting

One of the potential ways to impact a child's F/V intake is through exposure and experience with instructional gardens during childcare. Of the childcare providers interviewed for this study, more than half were currently using gardening activities in their facility. The other participants all expressed interest in implementing a gardening program in the future or had already taken preliminary steps in doing so.

Four of the childcare providers interviewed were using a formal gardening curriculum with their program. Other providers who had implemented gardening programs were not using any formal curriculum but instead relied on themselves or other staff members to create the activities. Several interviewees felt that there would be a great benefit of using a curriculum for both the children and staff. They were, however, unaware of curriculum resources available for this age group.

Themes associated with components necessary to initiate or improve instructional gardening programs included benefits and challenges as well as resource needs for gardening with young children. Benefits were separated into nutrition-related and non-nutrition-related benefits. Nutrition-related benefits included gardens providing healthy food for consumption at the childcare facility, increasing exposure to and willingness to try F/V among children, increased knowledge about food and where it comes from, and encouraging physical activity. One provider described the experience in this way: “they've been able to see every aspect of the growth process, even to consumption,” and “they were wanting to eat things that when they're presented at the table, they're not interested in.” Non-nutrition-related benefits that were discussed included gardens encouraging a connection to the natural environment, providing enjoyment for children, incorporating gardening activities into other areas of learning, teaching responsibility, and sharing the gardening experience with the families of the children. Providers described their experiences in the following ways: “it's a great math activity because you can graph it and you can put quantities to it…how many beans did they get today. You can do the science aspect, what do plants need to grow?” Another provider stated, “if you look at gardening from a sensory aspect… [children] need that tactile thing…so to feel the dirt, to feel the water, that's a great sensory activity.”

Challenges were also discussed with both participants who were currently gardening and those that were not. Common themes that arose in this category included design challenges, lack of resources such as curriculum, knowledge, financial support, and volunteer support, lack of staff support or participation, lack of time for gardening activities, and natural challenges such as weather, pest issues, and low garden yield. One participant described pest issues in this way, “we struggle a little bit with animal friends who also like our garden, but whereas we want the kids to be really hands-on with the garden, be able to actually get in there and pick, we don't want to surround the little garden with chicken wire.” Most participants felt they had enough space to accommodate their current or future gardening programs. The amount of space that each location had varied greatly since the settings ranged from semirural to suburban and urban areas. The few participants who reported not having a space for an outdoor garden still expressed interest in gardening activities such as seed sprouting and small container gardening activities in the classroom/childcare space.

Under the theme of resource needs, participants discussed the need for information about gardening, curriculum materials and lesson plans, community support, parent involvement and volunteers, funding, and gardening tools and equipment. One participant stated, “I don't know a lot about vegetable gardening. I'm learning. I've only done a couple of things. I would like to have maybe a book or something specifically on what to do for children.” In relation to funding support, some participants had been awarded grants, while others were interested in learning more about funds available to childcare providers for gardening. Still others, particularly family day care and group family day care providers, were using their own funds to supply materials for the gardening activities. The majority of the childcare providers said they had enough tools and equipment for a gardening program, but several would have liked to purchase more, particularly those appropriately sized for young children. The remaining participants did not currently have the necessary tools. One childcare provider mentioned that, with their gardening program, they use very few standard gardening tools but instead have the children dig, plant, and weed using their hands.

Participants who did not currently have gardening programs at their facility were asked about the factors preventing them from doing so. The factors discussed included lack of funding, time, and space for the garden.

## 4. Discussion

### 4.1. Dietary Intake of Preschool-Age Children in the Childcare Setting

The results of this study are consistent with current research on F/V consumption among preschool children. Fox et al. found that more than 25% of children between the ages of 2 and 3 years old were not consuming a distinct serving of vegetables at least once a day [9]. This is noteworthy because although many participants of this study were following the Child and Adult Care Food Program nutrition guidelines [19], overall, many children were not consuming recommended amounts of F/V (based on observations made by childcare providers). Many of the participants of this study expressed that children's willingness to try F/V ultimately dictated their consumption more than their access to them.

Consistent with Lynch and Batal's research [20], many participants felt that children were not necessarily eating very healthy at home and this was reflected in the child's willingness to try certain foods, particularly vegetables, in the childcare setting. Participants of the current study felt that it was their responsibility to make a positive impact on the dietary habits of children.

### 4.2. Benefits of Gardening in the Childcare Setting

The benefits of gardening with young children included both nutrition-related and non-nutrition-related benefits. Childcare providers felt that the children's knowledge regarding F/V, including how food is grown, was increased. Other researchers examining the knowledge of food origins among preschool children coupled with school gardening projects have supported these findings [4]. Many participants also described that, after gardening with children, they had an increased willingness to try F/V as well as an increased preference for them which is consistent with previous research [21, 22] and sheds light on practices that could aid children in consuming amounts of F/V closer to the recommendations. Although much of this research has focused on older children, the data collected from the current study make it possible to extend these findings to include preschool-age children.

As described by participants in this study, children taking part in gardening programs were able to share their experiences and increased knowledge with their families, which in some cases resulted in increased knowledge of parents, gardening at home, or participation in community gardens. Spears-Lanoix et al. found improved family eating behaviors in elementary school children participating in gardening interventions [7].

Non-nutrition-related benefits, including connections to the natural environment and sensory learning, were evident in this study. Some participants felt that gardening programs were beneficial as an interdisciplinary tool, particularly in a childcare setting, because there was more freedom in the curriculum than in later education settings.

Some existing research has focused on the benefit of gardening and other nature-based curriculum used with preschool-age children in science education and, specifically, process learning [23]. Several of the participants in this study noted that process learning is important during the early stages of a child's development and planting and caring for seeds is an excellent way to engage in that. Other ways in which childcare providers were able to extend the gardening experience into other areas of the curriculum were with simple math lessons and sensory learning activities. By using the gardening activities in all areas of the curriculum, these programs gain additional benefits and relevance outside of their impact on nutrition and health.

### 4.3. Challenges, Barriers, and Resources Needed for a Successful Gardening Program

The challenges and barriers discussed were similar to those experienced in gardening programs with older children as documented in previous research studies. These included an overall lack of resources, specifically a need for funding for materials and maintenance, training and education, curriculum resources, and volunteer support [11, 12].

Participants felt that a source of funding, particularly with initial costs, was necessary in order to obtain the materials for their gardening program to be successful. Grant funding and other financial assistance for gardening are available from a variety of sources; however, not all childcare providers in this study were aware of how to access it. In a summary report from the National Gardening Association, sources of possible funding for preschool gardening programs in addition to grants are described and include donations, school district funds, parent or volunteer organization funds, fundraisers, and childcare provider's personal funds [16]. Increasing awareness and access to financial resources among childcare providers is crucial to increasing the success and sustainability of gardening programs.

In addition to a need for financial resources, many participants felt they lacked much of the knowledge and support needed for a successful gardening program. These findings are consistent with those of Dobbs, Relf, and McDaniel, who examined the needs of elementary school teachers in implementing gardening programs. They found that a source of gardening information was critical to the success of a gardening program [12]. Providing information, training, and educational materials for staff members could possibly aid childcare providers in overcoming other barriers and challenges identified to garden implementation. Participants overwhelmingly stated they would be willing and interested to participate in further training to increase the impact and success of their gardening programs. Opportunities to partner with local gardening groups, Master Gardeners, extension programs, or other community organizations could provide an avenue of effective training to help childcare providers overcome the barriers they have experienced and have a more successful gardening program.

Another major resource lacking was curriculum materials. Although there are a number of gardening curricula in existence for use with preschool-age children [13, 14], it was apparent that many childcare providers were unaware of them. Curricula such as these encompass many of the aspects participants were concerned with including planning and organization of the activities, successfully carrying out the activities, and parent involvement.

Finally, childcare providers in this study described volunteers and community support as resources needed in implementing a successful gardening program. Previous research on successful community gardens describes partnerships between gardening programs and the communities as an effective means of gaining resources [24]. Partnering with local residents, neighborhood associations, or gardening groups to take on some of the responsibility for garden set-up and maintenance would be an avenue to consider in implementing instructional daycare gardens.

### 4.4. Strengths and Limitations

A strength of this research study includes the inclusion of a variety of childcare settings. Incorporating various types of childcare settings in this study allowed researchers to compare and contrast the factors affecting garden implementation in each type of setting. While many of the benefits and barriers were similar among the various settings, there were some that were unique to each type of facility. Additionally, since this study focused on a preschool-age population, it adds to the literature focusing on this age group. More research exists within school-age populations.

Limitations include the use of a structured interview instrument with participants. Although participants were asked to expand upon their responses and were allowed to provide additional detail to the questions asked, the use of a structured interview format limited the allowance for unanticipated responses by participants. A different recruiting method or larger sample size may have offered more diversity among participants. Among the sample population, there was sufficient geographic diversity, but little ethnic diversity. Additionally, socioeconomic data were not collected and may have been beneficial in determining factors influencing the benefits and barriers of gardening programs. Finally, participants whom agreed to take part in this study proved to be enthusiastic about gardening programs with young children. The lack of childcare providers in the sample population that had little or no interest in implementing a gardening program may have skewed the results of this study as this population may have offered further perceived barriers to instructional gardening activities.

### 4.5. Implications for Research and Practice

Opportunities for future research in the area of gardening programs with young children include quantitative studies to measure the impact of these programs, especially with respect to F/V intake. Like those examining the benefits of gardening with older children, measuring both nutrition-related parameters and social and academic data among young children would be helpful in quantifying the value of these programs. In order for childcare providers to be successful in their gardening programs, it is important for them to learn from the challenges of others and find ways to overcome these common barriers. Building strong connections between parents and childcare providers around child feeding and gardening may also be a means for improving the diet quality of children.

This research also shows that there is a need for increased awareness of existing resources related to instructional gardening to aid childcare providers, educators, and community members in implementing these programs. Increasing the connections between childcare providers and community resources would serve to enhance the success and sustainability of instructional gardening programs with the ultimate goal of improving the diet quality and health of children.

## 5. Key Messages

- The use of instructional gardens is a growing area of interest to enhance diet quality among children and limited research has been conducted with preschool-age children.
- Childcare providers are an important target group to focus on when advocating for the use of instructional gardens with young children.
- Providing childcare providers with the knowledge and resources to implement instructional gardens is an important component to achieving success with instructional gardening with young children.

## Figures and Tables

**Table 1:** Structured interview questions asked of childcare providers.

*Demographic question*:
What is your role here at the childcare center?
How many children are cared for here on a daily basis?
What is the age range?
How many care providers are employed at this site?

*Fruit and vegetable consumption*
How important do you feel fruit and vegetable consumption is for yourself?
How important do you feel fruit and vegetable consumption is for children?
What do you feel are some of the benefits of fruit and vegetable consumption for children?
Do you have any previous coursework or training in nutrition?
Do you feel that the children in your care are consuming the recommended amount of fruits and vegetables on a daily basis while at
your child care operation?
Do you feel that the children are willing to try the fruits and vegetables that you serve them?
What are some examples of the types of fruits and vegetables you serve?
Where do you get the fruits and vegetables you serve? For example, are you responsible for doing the shopping and if so, where do you
buy them If not, do you have a produce vendor?
Do you feel that the mealtime environment you provide in your childcare setting has an impact on the eating behaviors of the
children? Mealtime environment includes all of the factors involved during a meal such as timing, location, food served, and eating
behaviors of each individual participating in the meal.
What role do you feel you play in the child's eating behaviors?
Do you feel that the home environment influences the eating behavior of the children you care for?

*Gardening*
Do you currently use any gardening activities that would involve growing fruits or vegetables?
Do you use any formal gardening curriculum?
What do you feel are some of the benefits to having a garden in a childcare setting?
What are some of the factors that might prevent you from having a garden?
Do you have the space to implement a garden at your location?
Do you have the time necessary to implement and maintain a garden?
Do you have the tools, such as shovels, rakes, stakes, wire fencing, etc. to implement a garden or do you feel you would have access to
these supplies?
Do you think the children you care for would enjoy a gardening experience while at daycare?
Do you feel the parents of the children would be in favor of a gardening program?
Would you be willing to participate in gardening training?
What resources do you feel would be helpful in implementing more gardening activities here?

**Table 2:** Demographic information of childcare facilities.

Type of childcare setting	Number of children attending (range)	Age range of children	Number of childcare providers
Family day care	4–7	6 wks.–9 yrs	1
Group family day care	15	7 mos.–11 yrs	2
Day care center	18–134	6 wks–12 yrs	4–40
Head start program	143–368	6 wks–5 years	70
